# Association between haematological parameters and sickle cell genotypes in children with *Plasmodium falciparum* malaria resident in Kisumu County in Western Kenya

**DOI:** 10.1186/s12879-020-05625-z

**Published:** 2020-11-25

**Authors:** Paul Kosiyo, Walter Otieno, Jesse Gitaka, Elly O. Munde, Collins Ouma

**Affiliations:** 1 Department of Biomedical Science and Technology, School of Pub;ic Health and Community Development, Maseno University, Maseno, Kenya; 2 Department of Medical Laboratory Sciences, School of Medicine, Kisii University, P.O. Box 408-40200, Kisii, Kenya; 3 Department of Paediatrics and Child Health, School of Medicine, Maseno University, Private Bag, Maseno, Kenya; 4 School of Clinical Medicine, Mount Kenya University, Gen Kago Rd, P.O. Box 342 01000, Thika, Kenya; 5 Department of Clinical Medicine, School of Health Sciences, Kirinyaga University, P.O Box 143-10300, Kerugoya, Kenya

**Keywords:** Sickle cell, Genotype, Malaria, *Plasmodium falciparum*, Haemoglobin

## Abstract

**Background:**

Sickle cell disease (SCD) is a monogenic disorder due to point mutation in the β-globin gene resulting in substitution of Valine for Glutamic acid. The SCD is prevalent in *P. falciparum* endemic regions such as western Kenya. Carriage of different sickle cell genotypes may influence haematological parameter during malaria. Children resident in malaria holoendemic regions suffer more from malaria-related complications and this is moderated by the presence of the SCD. In the current study, we determined the association between sickle cell genotypes and haematological parameters in children with *P. falciparum* malaria resident in Kisumu County in Western Kenya.

**Methodology:**

Children (*n* = 217, aged 1–192 months) with acute febrile condition were recruited at Jaramogi Oginga Odinga Teaching and Referral Hospital. Chi-square (χ2) analysis was used to determine differences between proportions. Differences in haematological parameters were compared across groups using Kruskal Wallis test and between groups using Mann Whitney U test. Multivariate logistic regression analysis controlling for infection status was used to determine the association between sickle cell genotypes and haematological parameters.

**Results:**

Using HbAA as the reference group, multivariate logistic regression analysis revealed that carriage of HbSS was associated with reduced haemoglobin [OR = 0.310, 95% CI = 0.101–0.956, *P =* 0.041], reduced haematocrit [OR = 0.318, 95% CI = 0.128–0.793, *P =* 0.014], reduced RBC count [OR = 0.124, 95% CI = 0.045–0.337, *P =* 0.001], reduced MCHC [OR = 0.325, 95% CI = 0.118–0.892, *P =* 0.029], increased leucocytosis [OR = 9.283, 95% CI = 3.167–27.210, *P =* 0.001] and reduced monocytosis [OR = 0.319, 95% CI = 0.123–0.830, *P =* 0.019]. However, carriage of HbAS was only associated with increased micro-platelets [OR = 3.629, 95% CI = 1.291–8.276, *P* = 0.012].

**Conclusion:**

Results show that carriage of HbSS in children influence the levels of haemoglobin, haematocrit, RBC, MCHC, WBC and Monocytes. Therefore prior knowledge of HbSS should be considered to improve clinical management of haematological alterations during malaria in children.

**Supplementary Information:**

The online version contains supplementary material available at 10.1186/s12879-020-05625-z.

## Background

The sickle cell haemoglobin (HbS) is a genetic disorder due to a single nucleotide polymorphism (SNP) or a point mutation substituting thymine for adenine at the sixth codon of β gene, (6GAG > 6GTG). This leads to valine incorporation, rather than glutamine and results in haemoglobin tetramers (HbS) that aggregate into arrays upon deoxygenation in the tissues [1]. The resulting HbS forms long polymers in reduced oxygen tension due hydrophobic interaction of valine (at 85th position of the globin chain) and phenylalanine (at 88th position of the globin chain) [2]. The involved red blood cells (RBC) ultimately become less flexible since polymers lead to biochemical and rheological changes forming aggregates hence impair the blood flow causing vaso-occlusion which is the hallmark of this genetic disorder [3].

Sickle cell disease refers to a group of autosomal recessive genetic disorders characterized by the presence of HbS mutated allele of the globin chain, whereas the wild type is called the HbA allele [4, 5]. On the other hand, sickle cell trait (HbAS) is the heterozygous form of sickle cell disease where the individual is a carrier of the HbS (sickle cell) gene [4]. The global impact of sickle cell disease (SCD) has been estimated to be approximately 275,000 births per year [6] and it is equally approximated that it could reach 400,000 births by 2050 in regard to recent projections [7]. Africa is the most affected continent with sickle cell trait (SCT) prevalence being greater than 15% in Sub-Saharan Africa [8]. The geographical distribution of these SCD is very similar to that of malaria and it has been found that SCT confers some resistance to malaria [9, 10]. The aetiological agent of malaria is the protozoan organism, *Plasmodium* of which the main species is falciparum [11]. In areas of *P. falciparum* malaria holo-endemicity such as Kisumu in Western Kenya, malaria contributes to 63.4% of admissions in children less than 5 years [12]. Due to this protection associated with SCT, these disorders have reached a balanced polymorphism in malaria holo-endemic regions such as in Kisumu County in western Kenya [13, 14]. It is thought that SCD make the cell susceptible to haemolysis at reduced thresholds of oxygen and any interior or exterior stressors on the RBC can simply make them haemolyse [14]. Worse still, malaria is the most important parasitic disease of humans and claims the life of more children globally than any other infectious disease [15]. This has clearly led to the hypothesis that this mutation is driven a lot by the pressure resulting from malaria parasite [16–18]. Presently, it is well documented that *P. falciparum* is a driving force of evolution that has shaped the human genome and can select genes that contribute to resistance [16, 17]. Whenever a gene that has a potential for reducing fitness achieves high frequencies in the population, it is assumed that the gene confers a survival advantage [19]. Sufficient studies including the ones conducted in the same locality in Western Kenya and elsewhere, have demonstrated that individuals who are heterozygous for the HbS are protected from the severe form of malaria infection [9, 14, 20, 21]. Malaria confers a selective heterozygous survival advantage to carriers of the HbS gene and this is referred to as balanced polymorphism [18]. Based on previous studies, it is plausible that both *P. falciparum* malaria and sickle cell disease have independent significant impacts on the alteration of haematological parameters [21–24].

Haematological parameters have been used over the years as pointers in the diagnosis of both infectious and non-infectious diseases and more specifically haemoglobinopathies [25]. These haematological parameters include both blood cell indices such as mean cell haemoglobin (MCH), red cell distribution width (RDW) as well as mean cell haemoglobin concentration (MCHC) and primary red cell measurement such as red cell count, haematocrit and mean cell volume (MCV). Even though SCD is primarily a disease of red blood cells, both leucocytes and thrombocytes are equally affected [26]. The above-mentioned indices together with other parameters such as platelet count, mean platelet volume (MPV) and platelet distribution width (PDW) as well as leucocyte count are of major diagnostic significance and are equally important in the management of SCD and pathogenesis of the various sickle cell crises [3, 27]. It is therefore imperative to determine the association between the haematological parameters and sickle cell genotypes in *Plasmodium falciparum* malaria infection because these haematological parameters determine the occurrence of various sickle cell crises as demonstrated by vaso-occlusion [3, 27]. However, few studies in East Africa and none in Kenya has been undertaken to elucidate the association of these haematological parameters and sickle cell genotypes in *P. falciparum* infection [28]. Recently, a retrospective study involving patients with severe malaria in Cameroon did not find any significant association between these parameters and SCD [29], however, leucocytosis and thrombocytosis have been previously reported to drive sickle cell crises [27, 30]. To bridge this knowledge gap, our study aimed to determine the association between haematological parameters and sickle cell genotypes in children with *P. falciparum* infection enrolled at Jaramogi Oginga Teaching and Referral Hospital (JOOTRH), in Kisumu County within western Kenya.

## Methods

**Study Site:** The study was conducted at Jaramogi Oginga Odinga Teaching and Referral Hospital (JOOTRH) in Kisumu County between April 2018 and February 2019. JOOTRH is a level 5 hospital located within the headquarter of Kisumu County in Western Kenya and within Kisumu East Sub-County with a population of 150,124 in an area of 135.90 km2 [31]. This facility is the major referral health facility in Western Kenya for a greater population of 968,909 in an area of 2085.9 km^2^ [31]. Kisumu County is a malaria endemic area with prevalence at 27% [32]. Kisumu County has seven sub-counties namely Kisumu East, Kisumu West, Kisumu Central, Muhoroni, Seme, Nyando and Nyakach [31]. An earlier study in this site reported that the percentage of children aged below 5 years who had the SCT was 17.4% [13]. Furthermore, it has been confirmed that despite intensive malaria control approaches, this region show an increasing trend of malaria infection [33].

### Study population

This was a cross-sectional study targeting children aged 1–192 months and resident within Kisumu County. The sample size was calculated based on prevalence of sickle cell trait in the study area [13] using Cochran’s formula [34]. This period was selected since it captured the two major periods of the long and short seasonal rains from April to August and from November to December, respectively [35]. Furthermore, the period of enrolment was when the annual inoculation rates are usually high and estimated to be 300 infective bites per person per annum [35]. Throughout sampling, and even presently, there was neither routine screening for SCD in any place in Kenya nor any explicit nationally based guidelines regarding management of SCD. A validated structured questionnaire including demographic information, history of previous blood transfusion, and previous history of crises was administered prior to recruitment (See Supplementary File 1). All sickle cell patients were either on or not on routine tablets used in sickle cell clinics like vitamin B complex, paludrine and folic acids. No participant was on hydroxyurea therapy which could have impacted on the reported results for the sickle cell patient group.

### Inclusion criteria

Children in steady state and found to be infected with *P. falciparum* upon demonstration of asexual forms of *P. falciparum* through microscopic examination of both thick and thin smear with haemoglobin phenotype SS, AS and AA. Only participants who were HIV negative were included in the study. Besides meeting the above criteria, only children /patients whose parents were willing and able to provide written informed consent were included in the study.

### Exclusion criteria

Children with other forms of haemoglobinopathies e.g. thalassaemia syndromes. Participants with history of crises in the past 3 months, established by a careful history and complete physical examination as well as those with history of blood transfusion in the past 3 months were excluded. Those with acute bacterial, viral infections and parasitic infections other than *P. falciparum* and those with any form of malignancy were also excluded. Known sickle cell patients on hydroxyurea therapy were equally excluded.

### Ethical consideration

This study received ethical approval from Jaramogi Oginga Odinga Teaching and Referral Hospital Scientific and Ethics Review Committee (JOOTRH-ERC) - Approval NO. ERC.IB/VOL.1/414. An informed, written and voluntary consent was sought from the parents/guardians of children before obtaining blood samples for testing. Wherever applicable, children’s assent was also registered. The consent included strict confidentiality of the information generated during the study and their use for research.

### Laboratory procedures

#### Haematological measurements

Haemoglobin (Hb) levels and complete blood counts were determined using the Beckman Coulter ACT diff2™ (Beckman-Counter Corporation, Miami, FL, USA) in accordance to local Standard Operating Procedures (SOPs) within one hour of blood collection. Routine Quality Assurance checks were performed using commercial controls and recorded in conformity to manufacturer’s recommendations for the blood counts. The Analyser provided data on WBCs, RBCs, mean cell volume (MCV), haemoglobin level, platelet counts, mean platelet volume (MPV), platelet distribution width, (PDW) red cell distribution width (RDW) and three part differentials. The Analyser also detected and flagged platelet aggregation and cold agglutinins based on the particle size using a 256-channel pulse–height analyser of platelet histogram region.

#### Blood smears

Approximately 10 μl of blood was used for preparation of thick film while 2 μl of blood was used for thin smears. Thick smears were air-dried and stained with 10% Giemsa (Sigma-Aldrich, Germany) while thin smears were first fixed in absolute ethanol (Sigma-Aldrich, Germany) before staining with 10% Giemsa. After staining, a minimum of 50 high power fields (HPF) were scanned for a positive smear and 200 HPF for a negative by experienced Laboratory technologist. Diagnosis of malaria was based on the detection of asexual form of *P. falciparum* (trophozoite or schizont) of any density in the thick film. Thin blood films were further used for speciation of the *Plasmodium* species and for cytomorphological study of blood cell upon any flagging of haematological parameter in the complete blood picture due to platelet aggregation and cold agglutinins.

#### DNA extraction and quantification

Genomic DNA for sickle cell genotyping was extracted from 200 μL of whole blood. PureLink® DNA Mini Kit, (Invitrogen life technologies, USA) was used for DNA extraction following manufacturer’s instructions. The extraction procedure involved 4 major steps: Preparation of blood lysates, DNA binding, DNA washing and elution of 50 μL of DNA as per the manufacturer’s instructions. DNA quality and quantity was assessed using Nano Drop ND-1000 spectrophotometer (Thermofisher Scientific, San Diego, CO, USA) and stored at -20oC.

#### Sickle cell genotyping

Functionally tested TaqMan SNP Genotyping Assay was used in accordance with the manufacturer protocols (Life Technologies, Grand Island, NY). Identification of haemoglobin S was from biallellic discrimination (missense change [Glu6VAL) in the single nucleotide polymorphism rs334 by the following primer and probe sequences: Forward-TCAAACAGACACCATGGTGCAT, Reverse-CCCCACAGGGCAGTAACG, VIC-CTGACTCCTGAGGAGAA-MGB, 6FAM-CTGACTCCTGTGGAGAA-MGB, respectively. For quality assurance, a triplicate of control sample for genotype HbAA, HbAS and HbSS were run. Amplification was performed in Real-time PCR StepOnePlus thermo cycler from Applied Biosystems® (Foster City, CA, USA) through an initial denaturation at 95oC for 10 min, followed by 40 cycles of denaturation at 95oC for 1 min, annealing at 60oC for 1 min and final annealing at 60oC for 1 min.

### Statistical analyses

Statistical analyses were executed using Statistical Package for the Social Sciences (SPSS) version 22.0 software (IBM, New York, USA). Chi-square (χ2) analysis was used to determine differences between proportions. Mann-Whitney U test or student’s t-test was used for comparisons of demographic and laboratory characteristics between the clinical groups. Kruskal Wallis test was used to compare levels of haematological parameters across the haemoglobin genotypes. Association between haematological parameters and sickle cell genotypes was determined using multivariate logistic regression while association between sickle genotype and *P. falciparum* infection was determined by bivariate logistic regression. All statistical significance was set at *P* ≤ 0.05.

## Results

### Clinical, laboratory and demographic characteristics of study participants

Children (*n* = 217, aged 1–192 months) presenting with acute febrile conditions (temperature > 37.5oC) were recruited in the study. Out of these, 100 were females while 117 were males (See Supplementary File 2). A total of 148 (66 females and 82 males) had HbAA genotype, 45 (22 males and 23 females) had HbAS and 24 (13 females and 11 males) had HbSS genotype. The proportions of sex with regard to malaria infection between the clinical groups in each genotype stratum were comparable (*P =* 0.964, *P* = 0.921 and *P* = 0.167) for genotypes HbAA, HbAS and HbSS, respectively. Across group comparison on sex distribution demonstrated that this was also comparable across the genotypes (*P* = 0.880). Malaria of any parasite density, (asexual form of *P. falciparum* trophozoite or schizont) was microscopically confirmed in 58 HbAA, 14 HbAS and 13 HbSS with genotype-specific prevalence of 39.2% (58/148), 31.1% (14/45) and 54.2% (13/24), respectively. The remaining children (90 HbAA, 31 HbAS and 11 HbSS) were *P. falciparum* negative. In the HbAA group, children with *P. falciparum* infection group [median (IQR); 36 (63.3)] were older compared to those in non-infected group [median (IQR); 29.5 [36], *P =* 0.022]. Otherwise age did not differ between infected and non-infected groups both in HbAS [median (IQR); 36.3 (32.3)] and [median (IQR); 36 [37], *P =* 0.731] and HbSS [median (IQR); 31 (76)] and [median (IQR); 16 [27], *P =* 0.569], respectively. Haematological parameters were compared in the infected and non-infected children (controls) of the equal genotype (within the group) and in the infected children of different genotypes (across the group) i.e. HbAS versus HbSS. As shown in Table 1, haemoglobin (*P* = 0.001), haematocrit (*P* = 0.001) and RBC (*P* = 0.004) count were relatively lower in the infected group compared to non-infected group within each genotype. The RDW (*P* = 0.007) was moderately higher in the infected group compared to non-infected group in each stratum. Haemoglobin, haematocrit, RBC count and RDW were the most significantly different red cell parameter across the genotypes (*P* = 0.001, *P* = 0.001, *P* = 0.004 and *P* = 0.007, respectively). Furthermore, MCV (*P* = 0.332), MCH (*P* = 0.784) and MCHC (*P* = 0.179) were higher in the non-infected group relative to infected group in each genotype.

**Table 1:** Demographic and Laboratory characteristics of all the study participants

Characteristics	HbAA (***n*** = 148)			HbAS (***n*** = 45)			HbSS (***n*** = 24)			***P***-value+
	Malaria positive (*n* = 58)	Malaria Negative (*n* = 90)	*P*-value	Malaria Positive (n = 14)	Malaria Negative (*n* = 31)	*P*-value	Malaria Positive (*n* = 13)	Malaria Negative (*n* = 11)	*P* value	
**Sex, n (%)** Male	32 (55.2%)	50 (55.6%)	0.964a	7 (50%)	15 (48.4%)	0.921a	5 (38.5%)	8 (72.7%)	0.167a	0.880a
Female	26 (44.8%)	40 (44.4%)		7 (50%)	16 (51.6%)		8 (62.5%)	3 (27.3%)		
Age, (Months)	36 (63.3)	29.5 (37)	**0.022b**	36 (32.3)	36 (50)	0.731b	31 (76)	16 (27)	0.569b	0.507c
**Red cell parameters and its indices**										
Haemoglobin, gdL-1	10.2 (2.2)	10.5 (2.2)	0.190b	10.4 (1.6)	10.8 (2.3)	0.659b	7.3 (1.3)	9.2 (2.8)	**0.018b**	**0.001c**
Haematocrit, %	34.6 (8.3)	35.1 (5.7)	0.356b	32.9 (6.0)	36.8 (9.7)	0.185b	26.4 (4.4)	33.7 (6.8)	**0.002b**	**0.001c**
RBC, (× 10^12^ μL-1)	4.6 (1.0)	4.8 (0.7)	0.302b	4.8 (1.5)	5.0 (1.3)	0.532b	3.2 (1.7)	4.7 (1.2)	0.167b	**0.004c**
RDW, %	11.4 (2.2)	11.2 (2.2)	0.817b	11.5 (2.6)	11.7 (3.6)	0.589b	14.9 (3.3)	12.1 (10.3)	0.910b	**0.007c**
MCV, fL	75.1 (10.5)	75.7 (11.2)	0.327b	74.1 (11.8)	76.3 (17.3)	0.540b	79.5 (15.9)	76.8 (7.3)	1.000b	0.332c
MCH, fL/cell	22.15 (3.7)	22.9 (3.5)	0.277b	31.0 (1.7)	28.6 (3.2)	0.440b	23.1 (3.5)	22.5 (3.7)	0.569b	0.784c
MCHC, gdL-1	29.7 (2.2)	29.9 (4.1)	0.574b	31.0 (1.7)	28.6 (3.2)	**0.008b**	27.7 (4.1)	28.2 (2.4)	1.00b	0.179c
**Leucocyte parameters**										
WBC(×103 μL-1)	7.8 (4.8)	8.46 (5.5)	0.817b	9.58 (5.2)	8.3 (5)	0.433b	12.68 (4.6)	12.2 (7.0)	0.569b	**0.004c**
Lymphocytes, %	36.6 (27.2)	45.1 (24.3)	**0.050b**	37 (36.2)	43.6 (34.2)	0.844b	50.9 (29.1)	39.6 (17.8)	0.733b	0.873c
Monocytes, %	11.2 (7.4)	9.7 (4.6)	0.300b	12 (7.8)	8.4 (4.8)	0.140b	8.1 (6.1)	9.3 (3.5)	0.865b	0.866c
Granulocytes, %	51.5 (27.2)	45.6 (26.4)	0.186b	48.9 (27.5)	46.1 (28.1)	0.844b	39.3 (28.2)	51.1 (16.4)	0.649b	0.833c
**Platelet parameter and its indices**										
Platelet, (×103 μL-1)	220 (127)	271 (116)	**0.001b**	232 (129)	288 (102)	0.061b	236 (140)	265 (187)	0.955b	**0.001c**
MPV, fL	5.4 (0.6)	5.5 (0.5)	0.321b	5.3 (0.9)	5.5 (0.7)	0.258b	5.2 (0.8)	5.5 (0.6)	0.733b	0.710c
PDW, %	9.6 (1.4)	9.35 (1.5)	0.632b	9.5 (1.9)	9.5 (1.5)	0.556b	9.5 (0.4)	9 (1.5)	0.277b	0.611c
PCT, %	0.13 (0.1)	0.15 (0.1)	**0.003b**	0.1 (0.1)	0.2 (0.1)	0.102b	0.1 (0.1)	0.15 (0.1)	0.649b	0.260c

Data are presented as the median (interquartile range; IQR) values unless stated otherwise. Study participants were stratified into sickle cell genotype (HbAA, HbAS and HbSS). Each stratum was further categorised into infected and non-infected groups. a Statistical significance was determined by the Chi-square (χ2) analysis. b Statistical significance was determined using Mann Whitney test. c Statistical significance was determined using Kruskal Wallis test. *P* values in bold are statistically significant. Abbreviations: MCV; Mean corpuscular volume; MCH; mean corpuscular haemoglobin: MCHC; mean corpuscular haemoglobin WBC concentration; RBC-Red blood cells, RDW; Red cell distribution width; WBC; White blood cells, MPV; mean platelet volume; PDW; platelet distribution width and PCT. plateletcrit

Even though the WBC count were numerically lower in the infected group relative to non-infected group in HbAA genotype (*P* = 0.817) but higher in the infected group within both HbAS (*P* = 0.433) and HbSS (*P* = 0.569) genotypes, these differences were not statistically significant. However, the WBC count was statistically significant across the genotypes (*P* = 0.004). Lymphocyte was lower in the infected group relative to non-infected group within the HbAA genotype [median (IQR); 36.6 (27.2), and [median (IQR); 45 (224.3), *P =* 0.05], respectively. Both monocytes (*P* = 0.866) and granulocytes (*P* = 0.833) were comparable within the genotypes. Among the platelet parameters and its indices, platelet count was more exceptional in its comparability within the HbAA genotype. Infected group had lower platelet count relative to non-infected group [median (IQR); 220 (127), and [median (IQR); 271 (116), *P =* 0.001], respectively. This significance was equally simulated in the comparison across the three genotypes (*P* = 0.001). The MPV (*P* = 0.710) and PDW (*P* = 0.611) were comparable across the genotypes. The PCT was only statistically significant within HbAA genotype with [median (IQR); 0.13 (0.1), and [median (IQR); 0.15 (0.1), *P =* 0.003] for infected and non-infected groups, respectively. All the determined demographic and laboratory characteristics are summarized in Table 1.

### Association between haematological parameters and sickle cell genotypes in *P. falciparum* infected children

Considering the distribution of haematological parameters in the three stratified study groups, their association with sickle cell genotypes in *P. falciparum* infected children were modelled using logistic regression model while controlling for the infection status. Infection status was controlled for since sickle gene has an influence of the haematological parameters regardless of infection, however, malaria infection can further worsen the levels of these parameters. Using the homozygous wild-type genotypes (HbAA) as the reference group, results revealed no association between HbAS and haemoglobin [OR = 1.098, 95% CI = 0.555–2.171, *P =* 0.789], haematocrit [OR = 1.140, 95% CI = 0.568–2.287, *P =* 0.713], RBC count [OR = 0.706, 95% CI = 0.235–2.123, *P =* 0.100] and RDW [OR = 0.093, 95% CI = 0.514–2.323, *P =* 0.818]. However, there was a significant association between HbSS and haemoglobin [OR = 0.310, 95% CI = 0.101–0.956, *P =* 0.041], haematocrit [OR = 0.318, 95% CI = 0.128–0.793, *P =* 0.014], RBC count [OR = 0.124, 95% CI = 0.045–0.337, *P =* 0.001] and RDW [OR = 2.993, 95% CI = 0.952–9.409, *P =* 0.061] (See Table 2). Reduced MCV (microcytosis) was neither associated with HbAS [OR = 1.193, 95% CI = 0.609–2.334, *P =* 0.606] nor HbSS [OR = 2.094, 95% CI = 0.854–5.137, *P =* 0.106]. Furthermore, increased MCV (macrocytosis) [OR = 1.058, 95% CI = 0.107–10.452, *P =* 0.962] was not associated with HbAS, however, association between HbSS and increased MCV did not run in the regression model. Additional analyses revealed that reduced MCH did not show any association with HbAS [OR = 0.763, 95% CI = 0.263–2.219, *P =* 0.620]. Moreover, reduced MCH did not show any association with HbSS [OR = 0.507, 95% CI = 0.110–2.340, *P =* 0.383] while reduced MCHC did not reveal association with HbAS [OR = 0.819, 95% CI = 0.373–1.798, *P =* 0.619]. However, reduced MCHC showed association HbSS [OR = 0.325, 95% CI = 0.118–0.892, *P =* 0.029]. Total reduction in white blood cell count (leucocytopaenia) was not associated with HbAS [OR = 0.131, 95% CI = 0.011–1.528, *P =* 0.105]. However, leucocytosis showed increased association with HbSS [OR = 9.283, 95% CI = 3.167–27.210, *P =* 0.001]. Both elevated and reduced lymphocyte and granulocyte counts did not reveal association with either HbAS or HbSS in *P. falciparum* infected children. However, increased monocyte count (monocytosis) revealed association with HbSS [OR = 0.319, 95% CI = 0.123–0.830, *P =* 0.019].

**Table 2:** Association between haematological parameters and sickle cell genotypes

Parameter	Dichotomised haematological outcome	Sickle cell genotype	OR	95% CI	***P***-value
Haemoglobin	Anaemia (Haemoglobin < 10 gdL-1)	HbAS	1.098	0.555–2.171	0.789
		HbSS	0.310	0.101–0.956	**0.041**
Haematocrit	(Haematocrit < 33%)	HbAS	1.140	0.568–2.287	0.713
		HbSS	0.318	0.128–0.793	**0.014**
RBC count	Erythrocytopaenia (RBC count < 3.8 × 10^12^ μL-1)	HbAS	0.706	0.235–2.123	0.100
		HbSS	0.124	0.045–0.337	**0.001**
RDW	(< 12%)	HbAS	0.093	0.514–2.323	0.818
		HbSS	2.993	0.952–9.409	0.061
MCV	Microcytosis (MCV < 76 fL)	HbAS	1.193	0.609–2.334	0.606
		HbSS	2.094	0.854–5.137	0.106
	Macrocytosis (MCV > 96 fL)	HbAS	1.058	0.107–10.452	0.962
		HbSS	XXX	XXX	XXX
MCH	(< 25 Pg)	HbAS	0.763	0.263–2.219	0.620
		HbSS	0.507	0.110–2.340	0.384
MCHC	(< 28 gdL-1)	HbAS	0.819	0.373–1.798	0.619
		HbSS	0.325	0.118–0.892	**0.029**
WBC	Leucocytopaenia (WBC < 4 × 103 μL-1)	HbAS	0.131	0.011–1.528	0.105
		HbSS	XXX	XXX	XXX
	Leucocytosis (WBC > 10 × 103 μL-1)	HbAS	1.485	0.692–3.185	0.310
		HbSS	9.283	3.167–27.210	**0.001**
Lymphocytes	Lymphoctopenia (Lymphocytes < 20%)	HbAS	0.625	0.265–1.473	0.283
		HbSS	3.014	0.646–14.069	0.160
	Lymphocytosis (Lymphocytes > 40%)	HbAS	0.607	0.180–2.052	0.422
		HbSS	0.636	0.101–4.018	0.630
Monocytes	Monocytosis (Monocytes, > 10%)	HbAS	0.800	0.406–1.574	0.518
		HbSS	0.319	0.123–0.830	**0.019**
Granulocytes	Granulocytopaenia (Granulocytes, < 30%)	HbAS	0.446	0.182–1.089	0.760
		HbSS	2.201	0.459–10.547	0.324
	Granulocytosis (Granulocytes, > 70%)	HbAS	0.941	0.354–2.503	0.903
		HbSS	0.538	0.117–2.473	0.426
Platelets	Thrombocytopaenia (Platelet count < 150 × 103 μL-1)	HbAS	1.746	0.330–9.243	0.512
		HbSS	XXX	XXX	XXX
	Thrombocytosis (Platelet count > 300 × 103 μL-1)	HbAS	0.984	0.463–2.095	0.968
		HbSS	XXX	XXX	XXX
MPV	Micro platelets (Platelets < 6 fL) Megathrombocytes (platelets> 12.3 fL)	HbAS	3.269	1.291–8.276	**0.012**
		HbSS	4.781	0.355–6.470	0.238
		NA	NA	NA	NA
PDW	(PDW, < 6%)	HbAS	0.932	0.286–3.035	0.907
		HbSS	XXX	XXX	XXX
PCT	(PCT, < 0.22%)	HbAS	1.017	0.304–3.405	0.978
		HbSS	0.552	0.220–13.900	0.718

Children (n = 217) with acute febrile condition were categorized on the basis of sickle cell genotype (HbAA and HbSS). Odds ratios (OR) and 95% confidence intervals (CI) were determined using multivariate logistic regression analysis controlling for infection status. *P*-values in bold were statistically significant at *P* ≤ 0.05. XXX; did to run in the regression model. NA; not applicable. The reference groups in the logistic regression analysis were the homozygous wild-type genotypes (HbAA)

With regard to platelet parameters and its indices, reduced platelet count (thrombocytopenia) did not show association with HbAS [OR = 1.746, 95% CI = 0.330–9.243, *P =* 0.512]. Furthermore, increased thrombocyte count (thrombocytosis) equally showed no association with HbAS [OR = 0.984, 95% CI = 0.463–2.095, *P =* 0.968]. Unfortunately, the model could not determine association of either thrombocytopenia or thrombocytosis with HbSS genotype in children infected with malaria. Micro-platelet was defined as a platelet with reduced mean platelet volume (MPV) to below 6 fL [36]. Micro-platelet was associated with HbAS [OR = 3.269, 95% CI = 1.291–8.276, *P* = 0.012] but was no associated with HbSS [OR = 4.781, 95% CI = 0.355–6.470, *P =* 0.238]. Furthermore, PDW did not show any association with HbAS [OR = 0.932, 95% CI = 0.286–3.035, *P =* 0.907], however, its association with HbSS was undetermined. Finally, PCT did not show any association with HbAS [OR = 1.017, 95% CI = 0.304–3.405, *P =* 0.978] and with HbSS [OR = 0.552, 95% CI = 0.220–13.900, *P =* 0.718] (See Table 2).

### Association between sickle cell genotype and *Plasmodium falciparum* infection

With HbAA as the reference group, bivariate logistic regression analysis was modelled to determine association between sickle cell genotypes and *P. falciparum* infection. Results revealed that both HbAS [OR = 0.701, 95% CI = 0.344–1.429, *P =* 0.328] and HbSS [OR = 1.834, 95% CI = 0.774–4.369, *P =* 0.171], was not associated with *P. falciparum* infection (See Table 3).

**Table 3:** Association between Sickle cell genotype and *P. falciparum* infection

Sickle cell genotypes	***P. falciparum*** infection		
	OR	95% CI	***P***-value
HbAA, (n = 148)	Ref	–	–
HbAS, (n = 45)	0.701	0.344–1.429	0.328
HbSS, (n = 24)	0.1834	0.774–4.369	0.171

Children with acute febrile condition (n = 217) were grouped based on the haemoglobin genotype. Odds ratios (OR) and 95% confidence intervals (CI) were determined across genotypes using bivariate logistic regression model controlling for age, sex. *P*-values in bold was statistically significant at *P* ≤ 0.05. The reference groups in the regression analysis were the homozygous wild-type genotypes (HbAA)

## Discussion

Variations in blood cell counts and its parameters occur in congenital haematological disorders of which sickle cell disease is a prime example. Furthermore, such alterations have also been common in malaria, and reported to be more pronounced in *P. falciparum* infection for the HbAA genotype [21, 38]. However, the magnitude of such alterations differ depending on genetic, and epigenetic factors [22]. Our study provides a unique approach by not only looking at the comparisons of the haematological parameters in sickle cell genotypes in *P. falciparum* infection but also determines the association of these parameters to sickle cell gene in the infection. A few studies have been carried out in African countries where malaria is holoendemic with varied demonstrations on the protective effect of sickle cell gene (HbAS and HbSS) of asymptomatic and symptomatic *P. falciparum* infection of variable severity [14, 20, 22, 23]. None of the above studies have specifically described haematological differences on the basis of sickle cell genotypes in children presenting with steady state of *P. falciparum* malaria apart from Prohit et al. [24] and McAuley et al. [39]. However, Prohit et al used patients aged 15–34 years and narrowed it down to severe *P. falciparum* malaria as opposed to children with non-severe malaria in the current study. On the other hand, McAuley and colleagues [39] failed to determine a true association between the risks of severe malaria in HbSS due to low sample size (only 5 cases of HbSS were studied).

In our study, the prevalence of malaria was lower in HbAS (31.1%), in comparison to HbAA (39.2%). Likewise, numerous studies have demonstrated a lower prevalence of malaria among children with HbAS relative to those with HbAA [14, 40, 41]. Our finding is consistent with that of Makani et al. among other studies in Africa where sickle cell disease is prevalent and malaria is holoendemic [41]. Reduced prevalence to *P. falciparum* malaria in the HbAS genotype group could be attributed to the already postulated mechanisms which confer protective effect of haemoglobin S against *P. falciparum* malaria. They include [1] reduced ability of parasites to grow (reduced intra-erythrocyte growth of parasites [2, 42] accelerated sickling of infected RBCs and ultimate clearance [3, 43] increased humoral and cell-mediated immune response [44] and [4] aberrant cytoskeleton within erythrocytes which contains HbS and HbC results in defective trafficking and altered delay of parasite encoded protein-*Plasmodium falciparum* membrane protein 1 (PfEMP-1) on the surface of parasitized erythrocytes [45]. In addition, it has been demonstrated that tolerance phenomenon of host to *P. falciparum* infection resulting from HbS-induced chronic production of haeme-oxygenase-1 and carbon monoxide [46] and that [5] translocation of sickle cell erythrocyte microRNAs into *falciparum* inhibits parasite translocation [20]. Nonetheless, individuals of homozygous sickle cell genotype (HbSS) undergo severe clinical effects of malaria [47–49]. Increased MCV in children with HbSS as opposed to those with HbAA and HbAS shown in the current study could be attributed to the fact that children carrying the HbSS genotype have chronic haemolysis hence stimulating consistent haemopoiesis and haemopoietic processes geared towards compensatory responses and that young erythrocytes have higher MCV [37]. However, this hypothesis needs further explorations.

Leucocytosis has previously been reported in children infected with malaria [21, 50] and in children with sickle cell disease [30, 51] and both findings attribute this to active erythropoiesis. Nevertheless, thrombocytosis revealed in the current study may be explained by background haemolytic anaemia and auto-splenectomy [51].

To determine the associations of haematological parameters and sickle cell genotypes, anaemia was defined as a condition where the haemoglobin level was below the lower reference (< 10 gdL-1) and RBC count (< 3.8 ×  10^12^ μL-1) with regard to age, sex and altitude [36, 52, 53]. Association between reduced haemoglobin levels, reduced haematocrit and reduced RBC count with HbSS could be due to haemolytic mechanism and increased removal of parasitized red blood cells through erythrophagocytosis [38, 54–56]. Another factor that might lead to low levels of these haemoglobin parameters in children with HbSS is abnormally high tumour necrosis factor (TNF) in *P. falciparum* infection [56]. However this was not assessed in the context of the current study. In our study, carriage of HbSS was associated with reduced MCHC. This means that children with the carriage of HbSS and are infected with *P. falciparum* were less likely to have reduced MCHC. The mean corpuscular haemoglobin concentration (MCHC) was defined as a measure of the concentration of haemoglobin in a given volume of packed red blood cell with a reference range of 28–36 gdL-1 [53]. Reduced association of reduced MCHC with HbSS was not surprising due to the fact that MCHC is derived from haemoglobin concentration and haematocrit which are primary red cell measurement and any factor affecting haemoglobin and haematocrit in either sickle cell or malaria would definitely affect MCHC [25]. Leucocytosis was defined as the increase in the number of circulating total white blood cells to above 11 × 103 μL-1 of blood [36, 52]. Children of HbSS genotype with *P. falciparum* were nine times more likely to have leucocytosis. Increased association of leucocytosis with carriage of HbSS in the current study may still be attributed to increased erythropoiesis and consistent haemolysis [37] but more exploration on this is still wanting to establish weather carriage of HbSS is associated with left shift, right shift or mixed shifts leucocytosis. Furthermore, previous studies attributed leucocytosis to the pathogenesis of sickle cell crisis [27]. Monocytosis was defined as a haematological condition in which the monocyte count in peripheral blood if above 10% of the total leucocyte count [36]. The current study has additionally shown that carriage of HbSS is associated with reduced monocytosis. This finding has further reinforced that of Wongtong and colleagues [57] that investigated the association of absolute monocyte count with laboratory variables in patients with sickle cell disease. In this case, inflammatory state in carriage of HbSS may contribute to monocytosis [57]. Moreover, monocytes are activated in sickle cell disease with expression of tumour necrosis factor alpha (TNF-α) and interleukin-1 beta (IL-1β) as well as adhesion of molecule ligand, CD11b [58–60]. Association of reduced MPV with HbAS in children infected with malaria in the current study was quite amazing. Although, carriage of HbAS gene has been reported to offer protection against malaria morbidity and mortality [40, 61], a high or normal platelet count alongside micro-platelets often signifies inflammation, infection or cancer [62]. We the therefore attribute increase of these micro-platelets to malaria infection in the current study.

Numerous studies have demonstrated protective effect of HbSS from *P. falciparum* infection compared to HbAS but results in higher morbidity and mortality once the infection occur [14, 61, 63, 64]. However, we did not find any significant association between both HbAS and HbSS with *P. falciparum* infection*.* A study in Uganda involving both children and adults revealed that both HbAS and HbSS were associated with reduced non-severe malaria outcomes [28]. The difference in our results in reference to other studies could be attributed to the fact that we only used children (1–192 months) stratified into infected and non-infected groups. This is the first study in East Africa to elucidate the association between selected haematological parameters and haemoglobin genotypes in children with *P. falciparum*. However, our study is limited to the fact that we did not investigate the level of parasitaemia and other haemoglobinopathies such as Glucose-6-phosphate dehydrogenase (G6PD), and alpha thalassaemia which is known to have epistasis effects on sickle cell gene with respect to *P. falciparum* malaria [65]. A longitudinal study with a larger cohort of participants may be valuable in extrapolating the knowledge gap for determination and understanding of such associations.

## Conclusion

HbSS genotype is associated with low haemoglobin, haematocrit, RBC, MCHC, WBC and monocytes. In addition we show that, carriage of HbAS is associated with lower MPV in children infected with malaria. HbSS genotype is association with low haemoglobin, haematocrit, RBC, MCHC and moncytopenia. However, it is associated with increased leucocytosis in children with *P. falciparum* malaria. Moreover, our study revealed that carriage of HbAS is associated with lower MPV in children infected with malaria. Results from this study can help in the differential diagnosis of malaria infection in HbSS genotype based on haematological parameters in resource limited setting where sickle cell genotyping remains a challenge. Therefore, for proper management of *P. falciparum* malaria, sickle cell genotyping should be considered to guide on the administration of antimalarial prophylaxis to HbSS patients.

## Supplementary Information

**Additional file 1.**

**Additional file 2.**

## Data Availability

The datasets generated and/or analysed during the current study are not publicly available due to compliance with ethical reviewer’s guidelines but are obtainable from the corresponding author on reasonable request.

## Abbreviations

μg: Microgram
μl: Microlitre
CBD: Central Business District
EDTA: Ethylene diamine tetra-acetic acid
g/dl: Grams per decilitre
Hb: Haemoglobin
HbAA: Genotype of the normal haemoglobin
HbAS: Genotype in sickle cell trait
HbSS: Genotype in sickle cell anaemia
HIV: Human immunodeficiency virus
JOOTRH: Jaramogi Oginga Odinga Teaching and Referral Hospital
MCH: Mean cell haemoglobin
MCHC: Mean cell haemoglobin concentration
MCV: Mean cell volume
Mls: Millilitres
OPD: Outpatient Department
PBF: Peripheral blood film examination
PCR: Polymerase Chain Reaction
Pg: Picogram
RBC: Red blood cells
RDW: Red cell distribution width
RPM: Revolutions per minute
rs: Reference SNP number
SCA: Sickle cell anaemia
SCD: Sickle cell disease
SCT: Sickle cell trait
SPSS: Statistical package for social sciences
WHO: World Health Organisation
