# Supplementary material for: Association between haematological parameters and sickle cell genotypes in children with Plasmodium falciparum malaria resident in Kisumu County in Western Kenya
Source: BMC Infect Dis. 2020 Nov 25;20:887. doi: 10.1186/s12879-020-05625-z (PMC7690073; doi:10.1186/s12879-020-05625-z)
Supplement: Supplementary file 1 — Additional file 1. [file 12879_2020_5625_MOESM1_ESM.docx]

## Questionnaire

Study No......................... Date......................Name..........................................................

Age (Months)................ Sex: 1. Male 2. Female

County of Birth....................................... County of Residence:

**CLINICAL CHARACTERISTICS**

1. History of chronic disease 1.YES 2. NO 3. I don’t know
2. It the above question is YES the mention........................................................................................
3. Any drug given within the pas one month period 1. YES 2. NO
4. If the above question is YES, mention the drugs..............................................................................
5. Previous history of blood transfusion 1.YES 2. NO
6. If the above is YES, mention when the last transfusion was done…………………………………
7. Any illness experienced in the past two weeks 1.YES 2. NO
8. Nutritional status. 1. Malnourished 2. Not malnourished.

**Laboratory Data**

Rapid HIV Testing POS NEGATIVE

COULTERGRAM

Hb................. RBC................. PCV...............

MCV............ MCH................ MCHC...........

WBC............. GRA#…………

Plt.................... MPV................

PDW.............

PBF where applicable

Malaria parasites on thick smear: Trophozoites: YES/NO Schizonts: YES/NO Gametocytes: YES/NO

Thin smear for speciation: 1. *P. falciparum* 2. *P. malariae* 3*. P. ovale* 4. Mixed species.

Grading of parasitaemia...................................................

Sickle cell genotyping: 1. HbAA 2.HbAS HbSS

Lab Technologist’s signature………………..PI signature………………………….
